# Aggregation-Induced Emission (AIE) Polymeric Micelles for Imaging-Guided Photodynamic Cancer Therapy

**DOI:** 10.3390/nano8110921

**Published:** 2018-11-07

**Authors:** Yang Zhang, Cai-Xia Wang, Shi-Wen Huang

**Affiliations:** Key Laboratory of Biomedical Polymers of Ministry of Education, Department of Chemistry, Wuhan University, Wuhan 430072, China; zyxx2010@163.com (Y.Z.); 13554047782@163.com (C.-X.W.)

**Keywords:** polymeric micelles, aggregation induced emission, imaging, photodynamic, cancer therapy

## Abstract

Photodynamic therapy (PDT) is a noninvasive treatment for selectively killing malignant tumor cells. The photosensitizer is a necessary component of photodynamic nanomedicine. Many efforts have been made to develop new photosensitizers for efficient cancer photodynamic therapy. In this work, we report a novel nano photosensitizer, polymeric micelles (AIE-M) with aggregation induced emission characteristic, for photodynamic cancer therapy. AIE-M with sub-20 nm particle size is prepared by the self-assembly of salicylaldazine-incorporated amphiphilic polymer (AIE-1), which can produce reactive oxygen species (ROS) with light irradiation in solution. After uptake by cancer cells, AIE-M can specially sojourn in plasma membranes of cancer cells at the early stage and predominantly accumulate in the mitochondria of cancer cell at the late stage. The phototoxicity of AIE-M, resulting from the generation of intracellular ROS with light irradiation, can efficiently cause cancer cells death by apoptosis and necrosis. The advantages of AIE-M as a nano photosensitizer include the small size, highly colloidal stability in the process of preparation and storage, and high cell penetration. The ultra-low Critical Micelle Concentration (CMC) of AIE-1, negligible dark toxicity and super phototoxicity of AIE-M suggest its promising potential for image-guided PDT.

## 1. Introduction

Cancer has become a great threat to human health which is not only one of the main causes of human death, but also a great burden on people’s psychology. Besides the traditional methods widely used in the clinical cancer treatments, such as surgery, chemotherapy and radiotherapy, photodynamic therapy (PDT) is an alternative choice and has found an increasingly wide application in cancer therapy due to its high selectivity and minor trauma [1,2]. Light with an appropriate wavelength, photosensitizers (PSs) and oxygen are the three important elements of PDT, in which PSs are activated by light and then transmit energy to oxygen to produce reactive oxygen species (ROS) including singlet oxygen (^1^O_2_), causing cancer cell apoptosis and necrosis [3]. In addition, upon light illumination, the intrinsic fluorescence of PSs is often detected and significant for imaging-guided therapy [4].

**Scheme 1:**
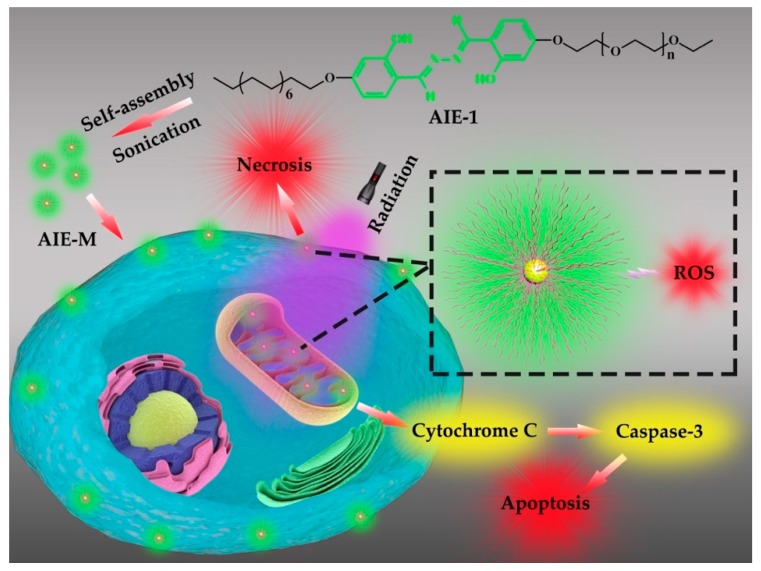
The illustration of AIE-M as a photosensitizer for imaging-guided cancer photodynamic therapy (PDT).

The development of PSs is one of the most important tasks in photodynamic therapy [5]. Several hundred compounds have been prepared and tested in photodynamic therapy, and some of them have been approved in the clinic [6,7]. Many of PSs are water insoluble and unsuitable for direct application in biological system. Nanotechnology provides a strategy to resolve the problem in the delivery of hydrophobic PSs [8,9,10,11]. However, the encapsulation of many hydrophobic PSs into nanocarriers often resulted in aggregation-caused quenching (ACQ) in fluorescence and decrease of ROS production, which are common in the case of porphyrin and other traditional PSs. The discovery of aggregation-induced emission (AIE) fluorogens can perfectly overcome the ACQ disadvantages of traditional fluorescent dyes [12,13,14,15]. Fluorescent imaging with AIE fluorescent probes has been recently widely applied in biological systems [16,17,18]. Furthermore, some AIEgens are found to efficiently generate ROS with laser irradiation in the aggregated state, and used as AIE PSs for imaging-guided PDT. Some AIE PSs showed both high fluorescence quantum yield and high ROS production efficiency in the aggregated state [19]. The nanosized particles formed from hydrophobic AIEgene or AIE PSs are often not colloidally stable in biological fluid. Two strategies have been developed to improve the colloidal stability of AIEgenes. One is to conjugate hydrophobic AIE units to hydrophilic polymers to form relatively large-sized nanoparticles [20]. Another is to encapsulate the AIEgens into polymeric micelles for cell imaging and/or photodynamic therapy [21]. We previously reported sub-20 nm AIE micelles (AIE-M) prepared from the assembly of a salicylaldazine-based amphiphilic polymer (AIE-1) (Scheme 1) [22]. The solution of AIE-1 in a good solvent shows no fluorescence, however, AIE-M in aqueous solution shows strong green fluorescence with laser irradiation. We further found the efficient production of ROS from AIE-M in aqueous solutions under light irradiation. In this work, we report the uptake of AIE-M by HeLa cells, intracellular generation of ROS induced by light, and photodynamic inhibition of HeLa cells growth.

## 2. Materials and Methods

### 2.1. Materials and Characterization

Cs_2_CO_3_, 1-bromohexadecane, 4-toluenesulfonyl chloride (TsCl), L-ascorbic acid (VC), hydrazine hydrate, ethanol, acetonitrile, methylbenzene, and dimethyl sulfoxide (DMSO) were purchased from Shanghai Chemical Co. (Shanghai, China) 2,4-dihydroxybenzaldehyde, 2,7-dichlorofluorescin diacetate (DCFH-DA), and poly (ethylene glycol) monomethyl ether (mPEG; Mn = 2000 Da) were obtained from Sigma-Aldrich (St. Louis, MO, USA). 1,3-diphenylisobenzofuran (DPBF) was purchased from Alfa Aesar (Ward Hill, MA, USA). Singlet oxygen sensor green (SOSG) was purchased from Invitrogen (Carlsbad, CA, USA). Dil, MitoTracker Red and Calcein-AM/PI dual staining kit were purchased from Beyotime Institute of Biotechnology (Shanghai, China). FITC Annexin V Apoptosis Detection Kit 1 was purchased from BD (San Diego, CA, USA). Thiazolyl Blue (MTT) and Trypan Blue was obtained from Beijing Dingguo Changsheng Biotechnology Co. (Beijing, China)

HeLa cells were purchased from the China Center for Type Culture Collection (Wuhan University, Wuhan, China) and cultured in Dulbecco’s modified Eagle medium (DMEM) supplemented with 4 mM L-glutamine, 10% fetal bovine serum (FBS) and 1% antibiotics (100 U mL^−1^ penicillin and 100 mg mL^−1^ streptomycin) at 37 °C in a humidified atmosphere containing 5% CO_2_.

^1^H and ^13^C NMR spectra were recorded on a Bruker Avance III HD NMR spectrometer (Karlsruhe, Germany). The morphology of AIE-M was observed using a TEM (H-7000FA, HITACHI, Tokyo, Japan). The measurements of micelles average sizes and polydispersity indexs (PDI) were carried out with a zetasizer (ZEN-3600, Malvern Instruments Ltd., Malvern, UK). Ultraviolet light is provided by a high-pressure mercury lamp with an ultraviolet filter (FC-100/FC, SPECTROLINE, Westbury, NY, USA). The fluorescence spectra were recorded on a spectrophotometer (RF-5301pc, Shimadzu, Kyoto, Japan). The absorption spectra were measured on a UV-Vis spectrophotometer (Lambda 35, Perkin Elmer, Waltham, MA, USA). The fluorescence lifetime was determined on a FELIX32 system (Photon Technology International, Birmingham, NJ, USA.). The micelles were prepared with a ultrasonic device (KQ218, Kunshan Ultrasonic Instruments Co., Kunshan, China). Cell photographs were taken by using a confocal laser scanning microscope (TE2000, EZ-C1, Nikon, Tokyo, Japan) and an inverted fluorescent microscope (IX70, Olympus, Tokyo, Japan). The cell fluorescence intensity was detected with a flow cytometer (CyAN-ADP, Beckman, Brea, CA, USA).

### 2.2. Synthesis of Compound *1*

1-Bromohexadecane (916 mg, 3 mmol) and 2,4-dihydroxybenzaldehyde (1050 mg, 7.5 mmol) were dissolved in acetonitrile (50 mL), then Cs_2_CO_3_ (977 mg, 3 mmol) was added. After refluxing at 85 °C for 24 h, the mixture was cooled down and filtrated to obtain yellow solution, which was concentrated to remove organic solvent and further separated by column chromatography (silica, petroleum ether:ethyl acetate = 10:1, *v*/*v*) to obtain compound 1 as white solid (708 mg, 65% yield). ^1^H NMR (CDCl_3_, 400 MHz): δ 11.49 (s, 1H), 9.71 (s, 1H), 7.42 (d, *J* = 8.68 Hz 1H), 6.53 (dd, *J*_1_ = 2.32 Hz, *J*_2_ = 2.28 Hz, 1H), 6.41 (d, *J* = 2.28 Hz, 1H), 4.00 (t, *J* = 6.6 Hz, 2H), 1.79 (m, 2H), 1.43–1.24 (m, 26H), 0.88 (t, *J* = 6.68 Hz, 3H). ^13^C NMR (CDCl_3_, 100 MHz): δ 194.30, 166.48, 164.54, 135.19, 114.99, 108.80, 101.04, 68.61, 29.70, 29.67, 29.58, 29.53, 29.37, 29.31, 28.92, 25.92, 22.70, 14.13.

### 2.3. Synthesis of Compound *2*

Hydrazine hydrate (300 mg, 6 mmol) was added to a two-necked flask which contained compound 1 (217 mg, 0.6 mmol) dissolved in 50 mL ethanol. The resulting mixture was refluxed under N_2_ atmosphere for 24 h, then cooled to 4 °C, and filtered. The light-yellow precipitates were washed with deionized water three times. After the solvent was removed by vacuum evaporation, the product of compound 2 was obtained as a yellow powder (177 mg, 72% yield). ^1^H NMR (CDCl_3_, 400 MHz): δ 11.27 (br, 1H), 7.84 (s, 1H), 6.99 (d, *J* = 8.52 Hz, 1H), 6.47 (d, *J* = 2.4 Hz, 1H), 6.42 (dd, *J*_1_ = 2.44 Hz, *J*_2_ = 2.44 Hz, 1H) 5.27 (br, 2H), 3.94 (t, *J*_1_ = 6.6 Hz, *J*_2_ = 6.6 Hz, 2H), 1.76 (m, 2H), 1.43–1.24 (m, 26H), 0.88 (t, *J* = 6.64 Hz, 3H). ^13^C NMR (CDCl_3_, 100 MHz): δ 160.97, 159.44, 147.36, 130.25, 111.69, 101.75, 68.07, 31.93, 29.70, 29.66, 29.60, 29.57, 29.37, 29.14, 26.01, 22.70, 14.13.

### 2.4. Synthesis of Compound *3*

Firstly, mPEGOTs was synthesized as an intermediate product. To remove the water from mPEG2000, mPEG2000 (10 g) was dissolved in toluene (75 mL) and refluxed at 140 °C for 4 h. After the mixture was cooled to room temperature, TsCl (1.25 g, 6.5 mmol) and TEA (750 mg, 7.5 mmol) were gradually dropped into the above solution with stirring in an ice bath. The reaction mixture was stirred for another 12 h and filtrated. The filtrate was concentrated to 20 mL, and mPEGOTs was obtained as a white precipitate while ethyl ether (500 mL) was added to the solution. Then, mPEGOTs (5 g), 2,4-dihydroxybenzaldehyde (350 mg, 2.5 mmol) and Cs_2_CO_3_ (815 mg, 2.5 mmol) were mixed in acetonitrile (50 mL) and stirred at 85 °C for 24 h. The mixture was filtered and concentrated to 20 mL. The precipitates were obtained by addition of ethyl ether (500 mL). The crude product was collected and purified by column chromatography (silica, dichloromethane:ethyl acetate = 10:1, *v*/*v*), and dried under vacuum to yield compound 3 as the white powder (3.58 g, yield 68%). ^1^H NMR (CDCl_3_, 400 MHz): 11.45 (s, 1H), 9.72 (s, 1H), 7.44 (d, *J* = 8.68 Hz, 1H), 6.57 (dd, *J*_1_ = 2.32 Hz, *J*_2_ = 2.36 Hz, 1H), 6.43 (d, *J* = 2.28, 1H), 4.18 (t, *J* = 4.64 Hz, 2H), 3.89-3.32 (m, 175H). ^13^C NMR (CDCl_3_, 100 MHz): δ 194.40, 165.94, 164.31, 135.23, 115.18, 108.68, 101.20, 71.86, 70.82, 70.50, 69.24, 67.85, 58.97.

### 2.5. Synthesis of AIE-1

Compound 2 (189 mg, 0.5 mmol) and compound 3 (1.18 g, 0.55 mmol) were dissolved in ethanol (20 mL) and stirred at 80 °C for 24 h. Then the mixture was cooled at 4 °C for 4 h and filtered. After removal of ethanol by vacuum evaporation, the yellow residue was dissolved in pure water and dialyzed against pure water (WMCO 3500 Da), and then lyophilized to afford AIE-1 as the yellow powder (874 mg, yield 70%). ^1^H NMR (CDCl_3_, 400 MHz): δ 8.61 (s, 2H), 7.23 (d, *J* = 8.36 Hz, 2H), 6.54 (m, 4H), 4.16 (t, *J* = 4.56 Hz, 2H), 3.99 (t, *J* = 6.56 Hz, 2H), 3.89-3.32 (m, 175H), 1.78 (m, 2H), 1.43–1.24 (m, 26H), 0.88 (t, *J* = 6.68 Hz, 3H). ^13^C NMR (CDCl_3_, 100 MHz): δ 163.46, 162.96, 162.87, 162.68, 161.71, 161.61, 133.45, 111.21, 110.85, 107.92, 107.89, 101.79, 101.61, 71.91, 70.85, 70.54, 69.46, 68.26, 67.60, 59.03, 31.90, 29.67, 29.63, 29.56, 29.53, 29.34, 29.04, 25.95, 22.67, 14.12.

### 2.6. Micelle Formation and Critical Micelle Concentration (CMC)

Due to the aggregation-induced emission characteristic of AIE-1, a simple method was developed to measure CMC by detecting the emergence of the fluorescence of AIE-M when it was assembled in water. A calculated amount of AIE-1 stock solution was added to pipes with different volumes of phosphate buffered saline PBS to obtain AIE-1 aqueous solutions at concentrations ranging from 0.02 to 12.8 μM. After 10 min of ultrasonic treatment, the fluorescence of the solutions was measured in 450–600 nm (Ex: 365 nm) with a fluorescence spectrophotometer. The fluorescence intensity at 525 nm was analyzed as a function of the polymer concentration. The CMC value was determined as the cross-point of the tangents with the two linear portions of the graph of the fluorescence intensity.

### 2.7. Preparation of Solution Containing Micelles (AIE-M)

AIE-1 (100 mg, 0.04 mol) was added to an aqueous solution (10 mL) in a 15 mL centrifuge tube, and then the AIE micelles (AIE-M) were prepared by ultrasonic treatment for 10 min and stored at 4 °C for further use. The power of ultrasonic device was 100 W and the frequency was 40 KHz.

### 2.8. Characterization of Micelles

The average sizes and polydispersity indexs (PDI) of AIE-M were determined by dynamic light scattering (DLS) using a Malvern Nano-ZS instrument (Malvern, UK) with a He-Ne laser beam (633 nm). The morphology of the nanoparticles was observed using a TEM at an acceleration voltage of 100 kV. Briefly, a drop of AIE-M solution was placed on a copper grid with formvar film and stained with a 0.2% (*w*/*v*) solution of phosphotungstic acid. For stability test of AIE-M, the PBS solution of AIE-M (1 mg/mL) was stored at 4 °C for 4 weeks. The average sizes and PDI of AIE-M were recorded at the scheduled time.

### 2.9. Photostability Assay

An aqueous solution of AIE-M (20 μM) in a cuvette was subjected to continuous UV irradiation (10 min, 10 mW cm^−2^ or 30 min, 1 mW cm^−2^). The absorption spectrum and the fluorescence intensity were measured using a UV-Vis spectrophotometer and a fluorescence spectrophotometer (Ex: 365 nm, Em: 525 nm), respectively.

### 2.10. Extracellular and Intracellular ROS Detection

1,3-Diphenylisobenzofuran (DPBF) was used to detect the generation of ROS from AIE-1 upon light irradiation in DMSO. Dichlorofluorescein (DCFH), an ROS indicator, was used to detect the ROS generation in PBS and cells. SOSG was used to detect the singlet oxygen. For the extracellular experiments, in the case of DMSO solution, the mixture solution of DPBF (100 μM) and AIE-1 (10 μM) in DMSO was exposed to 365 nm light irradiation (10 mW cm^−2^) and the absorbance of DPBF at 418 nm was monitored with a UV-Vis spectrometer. In the case of aqueous solution, DCFH stock solution (40 μM) in PBS was firstly prepared according to the reported procedure [23]. Then a mixture solution containing AIE-M (10 μM) and DCFH (10 μM) was irradiated with 365 nm light (10 mW cm^−2^). The fluorescence spectra were measured with a fluorescence spectrophotometer (Ex: 488 nm, Em: 525 nm). In parallel experiments, vitamin C (30 mM) as the ROS scavenger was added to the mixture before radiation The FL of DCFH solution upon radiation was also assessed as a control. For the detection of singlet oxygen, a mixture solution of AIE-M (10 μM) and SOSG (5 μM) was irradiated under the UV light (10 mW cm^−2^) for different time. The fluorescence spectra were measured with a fluorescence spectrophotometer (Ex: 488 nm, Em: 525 nm).

For detection of intracellular ROS generation, HeLa cells were seeded in glass bottom dishes at a density of 1.5 × 10^5^ cells per well and incubated for 24 h. Then HeLa cells were treated with AIE-M (20 μM) for 8 h in the dark. After removing non-internalized AIE-M, fresh DMEM containing DCFH-DA (20 μM) was added for another 30 min incubation. After being washed with PBS for 3 times, HeLa cells were irradiated with 365 nm light (10 mW cm^−2^, 150 s) and observed with a confocal laser scanning microscopy (Ex: 488 nm, Em: 515–545 nm). For flow cytometer analysis, HeLa cells were seeded in 6-well plates at a density of 2.5 × 10^5^ cells per well and treated with the same procedure above. After light illumination (10 mW cm^−2^, 150 s), HeLa cells were collected by trypsinization and resuspended in PBS. The intracellular ROS level, reflected by the fluorescence intensity in the cells, was analyzed by flow cytometer (Ex: 488 nm, Em: 510–550 nm). 

### 2.11. Cellular Uptake of AIE-M

Confocal laser scanning microscope CLSM was used to examine the intracellular uptake of AIE-M in a visually recognizable way. Briefly, HeLa cells were seeded in glass bottom dishes at a density of 1.5 × 10^5^ cells and cultured for 24 h. To study the time-dependent cellular uptake, AIE-M (20 μM) was incubated with HeLa cells for 2, 4, 8, 12, and 24 h. To study the concentration-dependent cellular uptake, the cells were treated with 10, 20, and 40 μM AIE-M for 4 h. Then the cells were washed three times with PBS and fixed with 4% (*w*/*v*) paraformaldehyde aqueous solution at room temperature for 15 min. Finally, the cells were imaged using a confocal laser scanning microscopy (Ex: 405 nm, Em: 515–545 nm).

For flow cytometry FCM analysis, HeLa cells were seeded in a 6-well plate at a density of 2.5 × 10^5^ cells per well and incubated for 24 h. Then the cells were treated with AIE-M (20 μM) for 2, 4, 8, 12, and 24 h. Subsequently, the cells were washed three times with PBS and harvested. The intracellular uptake of AIE-M, reflected by the fluorescence intensity in the cells, was detected by flow cytometry (Ex: 405 nm, Em: 510–550 nm).

### 2.12. Colocalization Staining

HeLa cells were seeded in glass bottom dishes at a density of 1.5 × 10^5^ cells. After 2 or 8 h incubation with AIE-M (20 μM), the cells were washed three times with PBS. Then the cell membrane was stained with Dil (10 μM) in PBS for 10 min, and the mitochondria were stained with Mito-Tracker Red (100 nM) in PBS for 15 min. The stained cells were washed with PBS and imaged with a confocal laser scanning microscopy (Dil, Ex: 543 nm, Em: 615–645 nm; Mito-Tracker Red, Ex: 543 nm, Em: 615–645 nm).

### 2.13. Endocytosis Inhibition Assay

HeLa cells were seeded in glass-bottom dishes at a density of 1.5 × 10^5^ cells per well and cultured for 24 h. For the evaluation of uptake efficiency at low temperate, the cells were treated with AIE-M (20 μM) for 4 h at 4 °C. To study the effect of different inhibitors on endocytosis of AIE-M, the cells were incubated with DMEM medium containing AIE-M (20 μM) and methyl-cyclodextrin (MβCD, 5 mM), sucrose (0.45 M), and cytochalasin D (5 μM) for 4 h at 37 °C separately. Then the cells were washed 3 times with PBS and fixed with 4% (*w*/*v*) paraformaldehyde aqueous solution at room temperature for 15 min. After replacing the solution with PBS, the cells were observed with CLSM (Ex: 405 nm, Em: 515–545 nm).

For FCM analysis of endocytosis inhibition, HeLa cells were cultured in 6-well plates at a density of 1.5 × 10^5^ cells per well for 24 h. Then, the cells were treated in the same procedure as above, ruined with PBS and harvested. The mean fluorescence intensity of AIE-M in cells was determined by FCM (Ex: 405 nm, Em: 510–550 nm).

### 2.14. In Vitro Dark Cytotoxicity

The in vitro dark cytotoxicity of AIE-M against HeLa cells was evaluated by the MTT assay. Briefly, HeLa cells were seeded in a 96-well plate at a density of 5.0 × 10^3^ cells/well and incubated for 24 h. Then, 100 μL solution of AIE-M in DMEM medium was added to the wells and the final concentrations of AIE-M ranged from 0.39 μM to 200 μM. After further 48 h incubation, 20 μL of MTT stock solution (5 mg mL^−1^ in PBS) was added to each well and cultured for another 4 h. Then the formazan blue crystal was dissolved by replacing the media with 150 μL of DMSO. The absorbance of the solution was measured using a microplate reader at 570 nm. Cell viability was expressed as follows:

Cell viability (%) = (*A*_sample_ − *A*_0_)/(*A*_control_ − *A*_0_) × 100%.

where *A*_sample_ is the absorbance of the cells treated with samples and MTT, *A*_control_ is the absorbance of control cells treated with MTT, *A*_0_ is the absorbance of the cells without MTT or other samples. The *A*_sample_, *A*_control_, and *A*_0_ were obtained after subtracting the absorbance of DMSO. Data are presented as average ± SD (*n* = 4).

### 2.15. In Vitro PDT

HeLa cells were seeded in 96-well plates at a density of 5.0 × 10^3^ cells per well. After 24 h incubation, the cells were divided into different groups to investigate the effect of preincubation time, AIE-M concentration and light dose on killing cancer cells. For studying the influence of preincubation time, HeLa cells were treated with AIE-M (20 μM) for 2, 4, 6, and 8 h, followed by irradiation for 150 s at the power destiny of 10 mW cm^−2^. For the evaluation of the effect of AIE-M concentration, the cells were incubated with 5, 10, 20, 30 and 40 μM AIE-M for 8 h and irradiated with UV light (150 s, 10 mW cm^−2^). Because the light dose could be adjusted by the irradiation time and power, the cells were cultured with AIE-M (20 μM) for 8 h and were then irradiated with identical power destiny (10 mW cm^−2^) for different times ranging from 1 to 5 min or irradiated with the power destiny increasing from 1 to 20 mW cm^−2^ for an equal time (150 s). Before being exposed to the irradiation, all the groups were washed twice with PBS to remove the excess of AIE-M and refilled with fresh medium. After the PDT treatment, all the groups were incubated for further 24 h in the dark. Then, cell viability values (%) were evaluated by the MTT assay as the above procedure.

### 2.16. Live-Dead Assay

Propidium iodide (PI) and calcein acetoxymethylester (calcein-AM) were used for the live-dead assay. HeLa cells were seeded into glass bottom dishes at a density of 1.5 × 10^5^ cells per well and incubated for 24 h. Subsequently, the cells were cultured in 1 mL DMEM medium containing AIE-M (10 μM, 20 μM and 40 μM) for 8 h. The cells without any treatment were prepared as the control. Then, the cells were rinsed with PBS and irradiated under the UV light (10 mW cm^−2^, 150 s). After another 7 h incubation, the cells were stained with working solution containing PI (4 μM) and calcein-AM (2 μM) at 37 °C for 20 min. After washing with PBS for 3 times, the cells were observed under a confocal laser scanning microscopy (Calcein-AM, Ex: 488 nm, Em: 515–545 nm; PI, Ex: 543 nm, Em: 615–645 nm).

### 2.17. Apoptosis Analysis

HeLa cells were seeded in a 6-well plate at a density of 1.5 × 10^5^ cells/well. After 24 h incubation, the cells were treated with AIE-M (10 µM and 20 µM) and incubated for another 8 h. Then, the cells were washed 3 times with PBS and irradiated upon the UV light (10 mW cm^−2^, 150 s) in the fresh culture medium. After the PDT treatment, the cells were incubated for a further 3 h at 37 °C in the dark. Subsequently, the cells were harvested in centrifuge tubes, washed twice with PBS and resuspended in 0.5 mL annexin-binding buffer. 5 µL of Annexin V-FITC and 10 µL of propidium iodide (PI) were added and incubated at room temperature for 15 min in the dark. Finally, the cells were analyzed by flow cytometry (FITC, Ex: 488 nm, Em: 510–550 nm; PI, Ex: 488 nm, Em: 603–623 nm).

### 2.18. Western Blotting Analysis

HeLa cells were seeded into 6-well plates at a density of 2.5 × 10^5^ cells/well and cultured for 24 h at 37 °C. The cells were incubated with AIE-M (10 μM, 40 μM) for 8 h. Then the cells were washed 3 times with PBS and irradiated with UV light (10 mW cm^−2^, 150 s). By 4 h after treatment, the cells were washed with TBS buffer and lysed in 50 μL RIPA buffer (1× PBS, 1% NP-40, 0.5% Na-deoxycholate, 0.1% SDS, 10 µg mL^−1^ PMSF, 2 µg mL^−1^ aprotinin, 100 mM Na-orthovanadate). The suspension was centrifuged at 12,000 rpm at 4 °C for 5 min, and the supernatant was collected. Protein concentrations were determined via Bradford assay. Then, 40 μg protein of each sample was resuspended in 50 µL 5× SDS sample buffer and boiled for 5 min and separated on a 10% SDS-PAGE (15 μL per lane). After electrophoresis, the proteins were transferred to a PVDF membrane (Millipore) by semi-dry transfer cell (Bio-rad). The membranes were blocked with PBS solution (containing 5% skimmed milk) at room temperature for 1 h and incubated with specific primary antibodies (1:3000 dilution) overnight at 4 °C, followed by the treatment with appropriate peroxidase-conjugated secondary antibodies (1:3000 dilution) for 30 min at room temperature. At last, the specific proteins were detected using enhanced chemiluminescence. Glyceraldehyde-3-phosphate dehydrogenase (GAPDH) was employed as a protein loading control.

### 2.19. Trypan Blue Dye Exclusion Assay

HeLa cells were seeded in a 6-well plate at a density of 2.5 × 10^5^ cells per well and incubated for 24 h. Then the cells were treated with AIE-M (20 μM) for 8h, washed and irradiated with UV light (10 mW cm^−2^, 150 s). The cells without any treatment were used as the control. At 3, 5 and 7 h after PDT treatment, HeLa cells were lightly counterstained with 0.04% trypan blue in PBS for 3 min. The control group and the cells treated with ultraviolet A (UVA) alone and AIE-M alone (20 μM, 8 h) were counterstained with 0.04% trypan blue 7 h after PDT. Then all the cells were gently washed three times with PBS and immediately observed under an inverted microscope with a 40× objection.

## 3. Results and Discussion

### 3.1. Preparation and Characterization of AIE Micelle (AIE-M) 

Amphiphilic salicylaldazine-containing polymer AIE-1 was synthesized according to the method we previously reported (Supplementary Scheme S1) [22]. Briefly, compound 1, prepared from 1-bromohexadecane and 2,4-dihydroxybenzaldehyde, was treated with hydrazine hydrate in ethanol under reflux to obtain compound 2. The condensation of compound 2 and compound 3, derived from mPEGOTs and 2,4-dihydroxybenzaldehyde in the present of Cs_2_CO_3,_ led to the formation of AIE-1. The chemical structures of the intermediates and targeting molecule AIE-1 were confirmed by ^1^H NMR and ^13^C NMR (Figures S1–S8).

**Figure 1:**
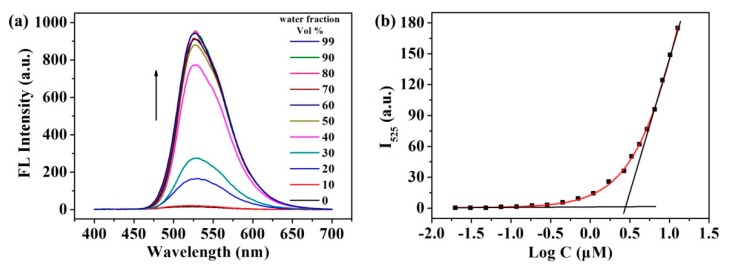
(**a**) Fluorescence spectra of AIE-1 in dimethyl sulfoxide (DMSO)/water mixtures; (**b**) Determination of the critical micelle concentration (CMC) of AIE-1.

AIE-1 shows typical aggregation-induced emission characteristics. The solution of AIE-1 in pure DMSO shows no fluorescence emission. The fluorescence emission at 525 nm was detected when water was added to the solution of AIE-1 in DMSO and the fluorescence intensity gradually increased while increasing the water fraction (*f*_w_) in the mixture of DMSO and water (Figure 1a).

As a classical method for the measurement of critical micelle concentration (CMC) of an amphiphilic polymer, pyrene is often chosen as a fluorescence probe. In the case of AIE-1, the salicylaldazine unit in AIE-1 was utilized as an intrinsic fluorescence probe and pyrene was deemed unnecessary to be added. CMC of AIE-1 was measured as low as 0.007 mg mL^−1^, which is comparable with cross-linkable polymer NPs and provides a prerequisite for biomedical applications (Figure 1b) [24].

**Figure 2:**
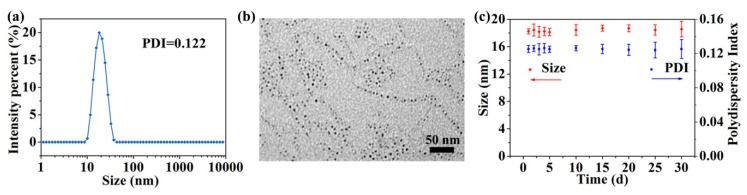
(**a**) The size distribution of AIE-M determined by dynamic light scattering (DLS) in water; (**b**) Transmission electron microscope (TEM) image of AIE-M after staining with 1% uranyl acetate; (**c**) Colloidal stability of AIE-M stored at 4 °C.

Due to its amphiphilic nature, AIE-1 could self-assemble into micelles (AIE-M). The nano micelles were prepared by simple ultrasonic treatment of AIE-1 solid in water. The average size of AIE-M, measured by dynamic light scattering (DLS) analysis, is 18 nm with a PDI of 0.122 (Figure 2a). The TEM image of AIE-M indicated that the micelles were spherical with good monodispersity and the diameters were around 6 nm in dry state (Figure 2b). AIE-M was highly colloidally stable in the process of preparation and storage. Even after storage at 4 °C for a month, there were no significant changes in the average sizes and PDI of AIE-M, which provided the unique advantage in biomedical application (Figure 2c).

**Figure 3:**
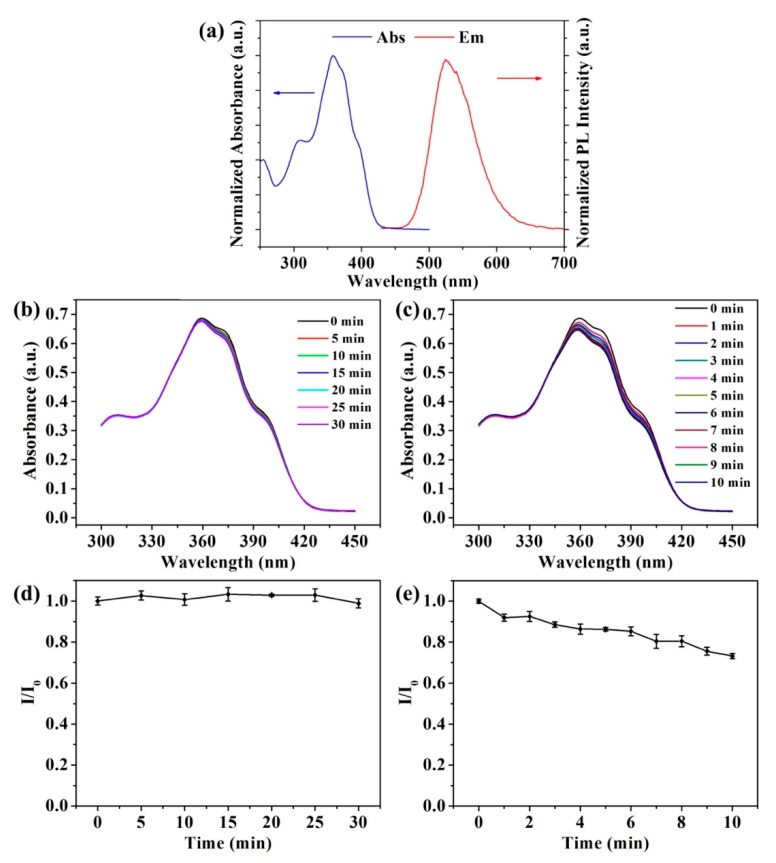
(**a**) Normalized ultraviolet-visible absorbance (blue) and PL (red) spectra of 20 μM AIE-M in water. The absorption spectra of AIE-M (**b**,**c**) and plots of *I*/*I*_0_ (**d**,**e**) upon continuous irradiation with 365 nm light at 1 mW cm^−2^ (**b**,**d**) or 10 mW cm^−2^ (**c**,**e**). *I*_0_ is the initial FL intensity of AIE-M, and *I* is the FL intensity of the sample after continuous irradiation for various time intervals.

The photochemical and photophysical properties of AIE-M were further investigated. The solution of AIE-M in water showed the absorption peak at 360 nm in the UV-Vis absorption spectrum and the maximum emission at 525 nm in PL spectrum with a large Stokes shift of 165 nm between the absorption and emission spectra (Figure 3a). AIE-M showed minimal self-absorption, which indicated its desirable fluorescence bioimaging potential. High photostability of AIE-M is another advantage in bioimaging. The significant change of fluorescence intensity of AIE-M in water at 525 nm was not observed after 30 min irradiation with 365 nm light (1 mW cm^−2^) (Figure 3b). The irradiation with higher-power light led to slight decrease of emission intensity (Figure 3c). 85% and 73% of the fluorescence intensity of AIE-M remained after 5 min and 10 min irradiation with 10 mW cm^−2^ light, respectively. In contrast, 365 nm light showed a minimal effect on the UV-Vis absorption of AIE-M (Figure 3d,e). In aqueous solution, the fluorescence lifetime of AIE-M was 1.442 ns (Figure S9) and the quantum yield was 0.14 [22].

### 3.2. Generation and Detection of ROS in Aqueous Solution

**Figure 4:**
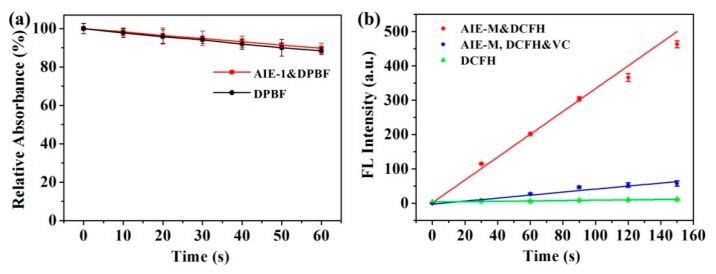
(**a**) The absorption changes at 418 nm of 1,3-diphenylisobenzofuran (DPBF) in DMSO and the mixed solution of DPBF and AIE-1 (10 μM) with 365nm light irradiation; (**b**) The FL intensity changes of Dichlorofluorescein (DCFH) solution and the mixture of DCFH and AIE-M (10 μM) in phosphate buffered saline (PBS) in the absence and presence of vitamin C (VC) after different time irradiation. The data are the mean values and standard deviations, n = 3.

The generation of ROS plays a vital role in causing critical damage to cancer cells in photodynamic therapy [25]. To evaluate the ROS generation ability of AIE-M in solution upon irradiation, 1,3-diphenylisobenzofuran (DPBF) and 2′,7′-dichlorodihydrofluorescein (DCFH) were used as the ROS indicators in DMSO and PBS, respectively. DPBF can be bleached by the oxidation with singlet oxygen, indicating the generation of ROS by the decrease of the absorbance of DPBF in the solution [26]. Non-fluorescent DCFH, an off–on probe of ROS, can transform into fluorescent dichlorofluorescein (DCF) through the oxidation of ROS [27]. As shown in Figure 4a, upon irradiation for 60 s, the 418 nm absorbance of the solution of AIE-1 and DPBF in DMSO decreased slightly, same as that of DPBF alone, suggesting no generation of ROS by fully dissolved AIE-1 solution. Interestingly, unlike the solution of AIE-1 in DMSO, AIE-M can efficiently generate ROS in aqueous solution upon light irradiation (Figure 4b). Before light irradiation, DCFH-containing AIE-M aqueous solution showed no fluorescence at 525 nm, indicating no production of DCF when DCFH and AIE-M was mixed. With 365 nm light irradiation, the aqueous solution of DCFH showed no fluorescence, ruling out the possible conversion of DCFH to DCF in the presence of light. In contrast, fluorescence was detected from the solution of AIE-M and DCFH after light irradiation, demonstrating the production of ROS. The fluorescence intensity at 525 nm approximately linearly increased with increasing the light irradiation time. When excessive vitamin C was added to the solution of AIE-M and DCFH, light irradiation generated ROS was almost completely scavenged by vitamin C. The fluorescence intensity at 525 nm significantly decreased. To further characterize ROS species generated in this system, singlet oxygen sensor green (SOSG) probe was used to detect the singlet oxygen [28]. When SOSG was mixed with AIE-M, the emission increased gradually with irradiation, demonstrating the production of singlet oxygen as the main cause of the cytotoxicity in Type II PDT (Figure S10). The ability of AIE-M with light irradiation to generate ROS predicts the potential in photodynamic therapy.

### 3.3. Cellular Uptake and Intracellular Localization

**Figure 5:**
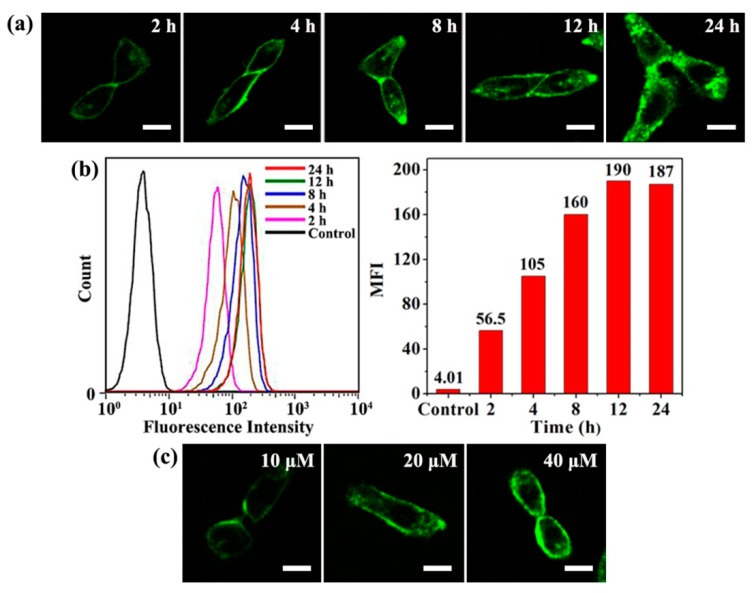
(**a**) Confocal laser scanning microscope (CLSM) images of HeLa cells treated with AIE-M (20 μM) for different incubation time; (**b**) Flow cytometry analyses of HeLa cells incubated with AIE-M for various incubation time; (**c**) CLSM images of HeLa cells incubated with 10 μM, 20 μM and 40 μM AIE-M for 4 h. The scale bar is 20 μm.

Efficient cellular uptake of the photosensitizer is essential for generation of intracellular ROS and subsequent PDT efficacy. The uptake of AIE-M with green fluorescence by HeLa cells was qualitatively and quantitatively analyzed by CLSM and FCM, respectively. We first evaluated the effect of incubation time, ranging from 2 h to 24 h, on the uptake of 20 μM AIE-M by HeLa cells. CLSM images in Figure 5a suggested that intracellular green fluorescence from AIE-M became stronger with an increase of incubation time from 2 h to 12 h, which implied a gradual increase of AIE-M uptake with increasing incubation time. No significant increase of AIE-M uptake by HeLa cells was observed when prolonging incubation time by 12 h. Quantitative FCM analysis results (Figure 5b) were consistent with that of CLSM observation. The increase of fluorescence intensity of HeLa cells incubated with AIE-M was rapid in the initial 8 h and became slow in the following 4 h, while no increase was found when incubation time increased from 12 h to 24 h. The concentration of AIE-M in the culture medium also affected the uptake by HeLa cells. As shown in Figure 5c, after 4 h incubation, the green fluorescence in HeLa cells, treated with 40 μM AIE-M, was higher than that in HeLa cells treated with 10 μM and 20 μM AIE-M-containing medium. The above results demonstrated that the uptake of AIE-M by HeLa cells is both time- and concentration-dependent. AIE-M can efficiently enter into HeLa cells after 8 h of incubation. 

**Figure 6:**
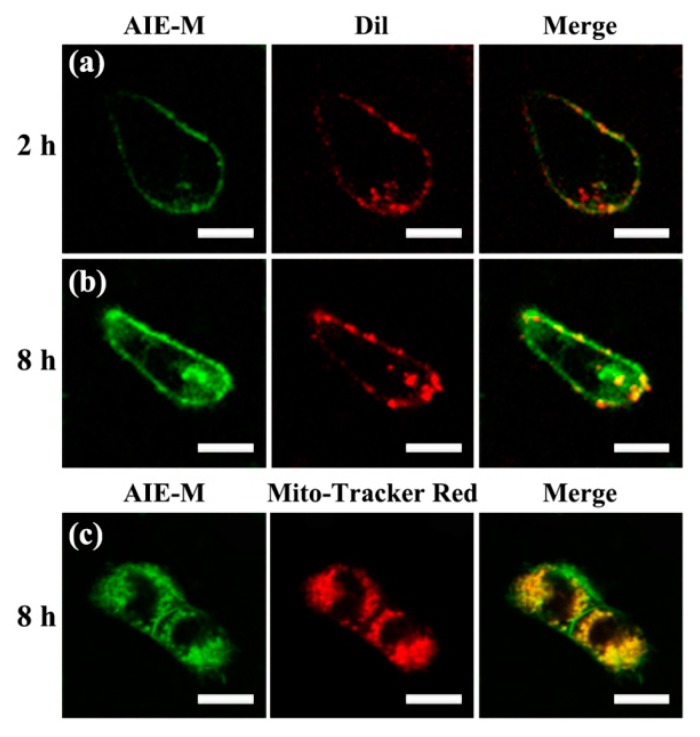
CLSM images of HeLa cells separately incubated with AIE-M (20 μM) for 2 h (**a**) or 8 h (**b**,**c**). HeLa cells were separately co-stained with 10 μM Dil (**a**,**b**) or 200 nM Mito-tracker Red (**c**). The scale bar is 20 μm.

The internalization and localization of the photosensitizer after cellular uptake is important for efficient cancer PDT. The membrane and mitochondrial localization of AIE-M in cells after 2 h and 8 h incubation was separately observed with CLSM using Dil and Mito-Tracker Red as membrane and mitochondrial co-localization dyes, respectively. As shown in Figure 6a, in the early stage (after 2 h incubation), the green fluorescence from AIE-M showed excellent overlap with the red fluorescence from Dil. The results indicated that AIE-M mainly localized on HeLa cell membranes, which might be ascribed to the steric effect provided by the PEG molecules retarding the transmembrane process and leading to the localization of AIE-M on the cell membrane at the early stage of incubation [29]. After 8 h of incubation, AIE-M in HeLa cells was divided into two parts. One part of AIE-M localized on the cell membrane, while another part localized in the mitochondria of HeLa cells. Beside the overlap of green fluorescence from AIE-M with red fluorescence from Dil (Figure 6b), significant overlap of green fluorescence from AIE-M with red fluorescence from Mito-Tracker Red was also observed (Figure 6c). Since both cell membrane and mitochondria are important targets of PDT, the localization of AIE-M in HeLa cell membranes and mitochondria after uptake, and local production of ROS from AIE-M with light irradiation are predicted to directly destroy cancer cell membrane and mitochondria, leading to efficient photodynamic therapy [30].

### 3.4. Endocytosis Inhibition

**Figure 7:**
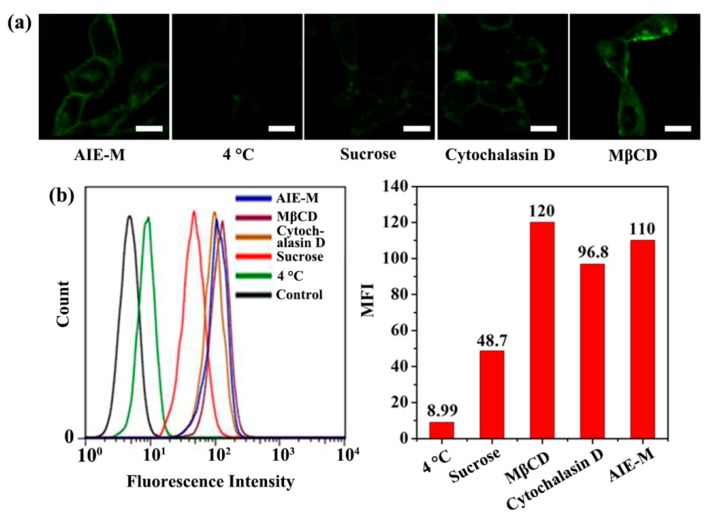
(**a**) CLSM images of HeLa cells after incubation with AIE-M containing different endocytosis inhibitor. The scale bar is 20 μm; (**b**) Flow cytometry analyses of the effect of endocytosis inhibitors on the uptake of AIE-M by HeLa cells.

Nano micelles often enter cells through an endocytosis mechanism. The endocytosis pathways of AIE-M by HeLa cells were investigated using CLSM and FCM. Low temperature (4 °C) could remarkably reduce the active uptake of nanocarriers. HeLa cells were separately treated with 20 μM AIE-M for 4 h at 4 °C and 37 °C, and observed with CLSM. The CLSM images, shown in Figure 7a, indicated that the AIE-M green fluorescence in cells after treatment at 4 °C was much weaker than that at 37 °C, suggesting that the uptake pathway of AIE-M by HeLa cells was energy-dependent endocytosis. Three different endocytosis inhibitors, cytochalasin D, MβCD and hypertonic sucrose, were used to evaluate the effects on the uptake of AIE-M by HeLa cells. Cytochalasin D inhibits macropinocytosis, while MβCD inhibits caveolae-mediated endocytosis and hypertonic sucrose inhibits clathrin-mediated endocytosis [31]. AIE-M green fluorescence in HeLa cells separately treated with 20 μM AIE-M in the presence or absence of endocytosis inhibitors was observed with CLSM (Figure 7a). It was found that hypertonic sucrose in cell culture medium significantly decreased the green fluorescence strength in comparison to the control without addition of the endocytosis inhibitor. In contrast, cytochalasin D and MβCD showed no significant effect on the fluorescence strength in HeLa cells treated with AIE-M together with cytochalasin D or MβCD. The endocytosis inhibition was further quantitatively evaluated using flow cytometry analysis. The quantitative analysis results, shown in Figure 7b, were completely consistent with CLSM observation. Both CLSM observation and FCM analysis results supported that clathrin-mediated endocytosis, rather than macropinocytosis and caveolae-mediated endocytosis, was the primary pathway for AIE-M to enter HeLa cells.

### 3.5. Production of Intracellular ROS 

**Figure 8:**
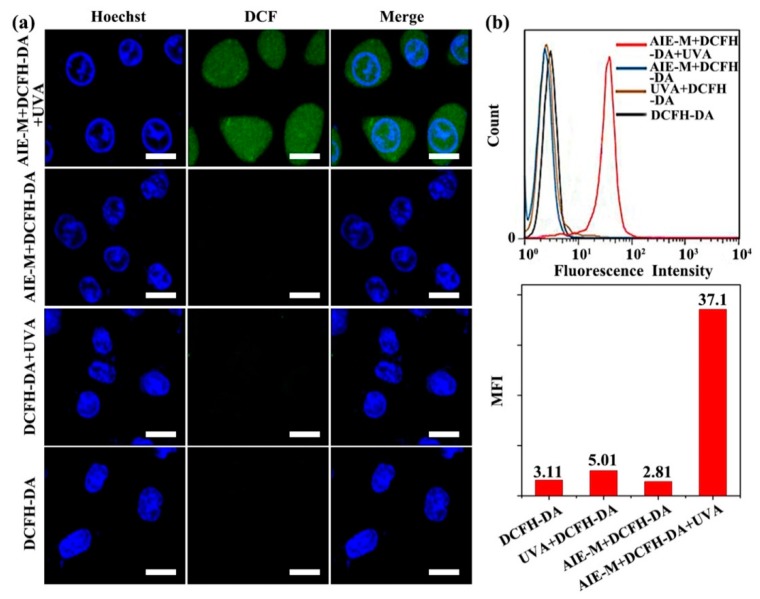
(**a**) CLSM images of intracellular ROS generation in HeLa cells co-stained with Hoechst 33342. The scale bar is 20 μm. (**b**) Flow cytometry analyses of ROS generation in HeLa cells under UVA irradiation.

The generation of ROS in HeLa cells treated with AIE-M and 365 nm light irradiation was monitored using DCFH-DA as the indicator. HeLa cells were incubated with 20 μM AIE-M for 8 h, washed and further incubated with DCFH-DA. The cells were irradiated at a light dose of 1.5 J cm^−2^. After cellular uptake, DCFH-DA was converted into DCFH inside the cells, which is non-fluorescent and reacted with ROS generated in the cells, leading to a green fluorescence product DCF. As shown in Figure 8a, in the case of HeLa cells separately treated with DCFH-DA, light and AIE-M, almost no green fluorescence was observed, indicating no sufficient ROS generation in the cells with separate treatments of the probe, light/probe and the micelles/probe. In the case of HeLa cells treated in proper order with AIE-M, DCFH-DA and light, strong green fluorescence was observed in the cells, demonstrating that the combined treatment of HeLa cells with AIE-M and light could efficiently generate intracellular ROS. Quantitative measurement of ROS generation was further conducted with flow cytometry analysis (Figure 8b). The FCM analysis results were consistent with CLSM observations. The ability of AIE-M with light irradiation to produce sufficient ROS in HeLa cells suggested the possible application in cancer PDT.

### 3.6. In Vitro PDT

**Figure 9:**
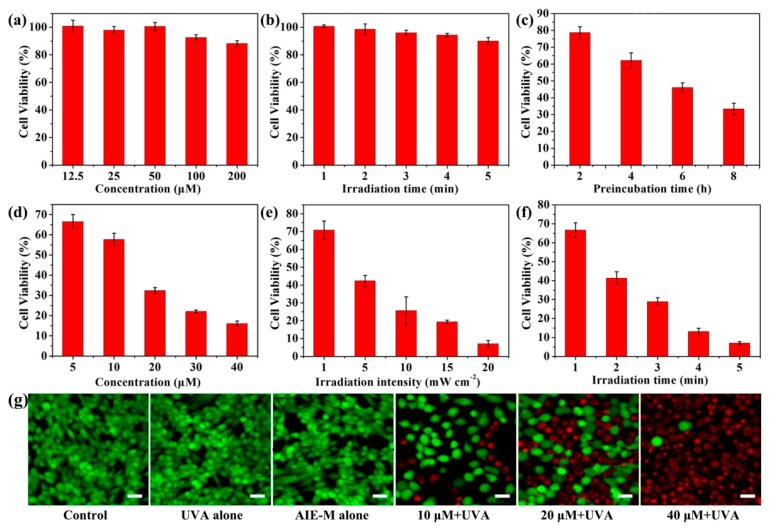
Viability of HeLa cells treated with AIE-M at variable concentrations for 48 h without light irradiation (**a**), light irradiation only (10 mW cm^−2^) for different irradiation times (**b**), AIE-M (20 μM) for different preincubation time and light irradiation (1.5 J cm^−2^) (**c**), AIE-M at different concentrations (5–40 μM) for 8 h and light irradiation (1.5 J cm^−2^) (**d**), AIE-M (20 μM) for 8 h and different power of light irradiation (1–20 mW cm^−2^) (**e**), AIE-M (20 μM) for 8 h and different light irradiation time (1–5 min) (**f**). CLSM images of HeLa cells stained with calcein-AM and PI after various treatments (**g**). The scale bar is 40 μm.

MTT assay was utilized to evaluate the cytotoxicity of light and AIE-M in the absence or presence of light. The low dark toxicity of the photosensitizer or photosensitizer-containing nanomedicine is the first prerequisite for successful cancer PDT. Prior to PDT evaluation, HeLa cells were incubated with AIE-M at various concentrations for 48 h. The results in Figure 9a indicated that AIE-M was almost nontoxic against HeLa cells at a concentration of 50 μM, and showed very low toxicity at concentrations up to 200 μM. To avoid the possible dark toxicity from AIE-M, AIE-M at a concentration less than 50 μM was selected for PDT in the next step; 365 nm light irradiation, ranging from 0.6 J cm^−2^ (1 min, 10 mW cm^−2^) to 3.0 J cm^−2^ (5 min, 10 mW cm^−2^), also showed negligible toxicity to HeLa cells under the experimental conditions in this work (Figure 9b).

In the case of photocytotoxicity of AIE-M, prior to light irradiation, HeLa cells were pre-incubated with AIE-M for 8 h. The cells were washed, irradiated with 365 nm light, and further incubated in fresh complete DMEM for another 24 h. To obtain optimized efficiency of cancer PDT, we studied the effects of AIE-M uptake time, AIE-M concentration, light irradiation intensities and light irradiation time on the growth of HeLa cells. As shown in Figure 9c, the pretreatment time of HeLa cells with AIE-M before light irradiation, namely uptake time, greatly affected PDT efficiencies. When AIE-M concentration and light dose were separately fixed at 20 μM and 1.5 J cm^−2^, respectively, the survival rate of HeLa cells decreased from 79% to 33% while the uptake time increased from 2 h to 8 h, which were consistent with the above cellular uptake results (Figure 5a) and demonstrated the importance of AIE-M cellular uptake for successful PDT. We fixed the uptake of 8 h in the following PDT experiments. The concentration of AIE-M in pretreatment medium is another important factor to affect PDT efficiency. With light irradiation at 1.5 J cm^−2^ (150 s, 10 mW cm^−2^), the viability of HeLa cells was AIE-M-dose-dependent, decreasing from 66% to 15% with the increase of AIE-M concentration from 5 μM to 40 μM (Figure 9d). Light dose also showed a great effect on the phototoxicity of AIE-M. Fixing the concentration of AIE-M at 20 μM and uptake time at 8 h, the increase of light dose led to an increase in phototoxicity of AIE-M. Both increasing light intensity and prolonging light irradiation time enhanced PDT efficiencies, however, the same total light dose, same AIE-M concentration and same uptake time resulted in almost the same phototoxicity. For example, the viabilities of HeLa cells induced by 3.0 J cm^−2^ (150 s, 20 mW cm^−2^) irradiation and 3.0 J cm^−2^ (300 s, 10 mW cm^−2^) were almost the same as 7% (Figure 9e, f). These results suggested the promising potential of AIE-M in cancer PDT.

The phototoxicity of AIE-M was further evaluated with live–dead cell staining. Calcein-AM is non-fluorescent and can permeate the cell membrane. Once inside the cells, it can be hydrolyzed by the cellular esterase and converted into fluorescent calcein, imaging the live cells in green fluorescence. Propidium iodide (PI) dye can only enter the dead cells or late apoptotic cells with damaged membrane and stain the cell nucleus, showing a bright-red fluorescence signal. Only green fluorescence was observed in HeLa cells without any treatment, treated with light irradiation only or AIE-M only (Figure 9g). The results demonstrated that both light irradiation and AIE-M without light irradiation are almost nontoxic to HeLa cells. However, the red fluorescence, representing dead or late apoptotic cells, appeared after HeLa cells were treated with the combination of AIE-M and light irradiation. The number of dead or late apoptotic cells increased with increasing AIE-M concentration from 10 to 40 μM (Figure 9g), which was consistent with the MTT assay results. Most HeLa cells were dead after co-treatment with 40 μM AIE-M and 1.5 J cm^−2^ light irradiation (150 s, 10 mW cm^−2^).

### 3.7. Cell Death Pathway

The death pathway of HeLa cells triggered by ROS generated from AIE-M upon irradiation was further detected. It is known that photodynamic generated intracellular ROS, mainly singlet oxygen, can only travel for a limited distance (~100 nm) in the biological system. It is generally considered that the primary damage occurs at intracellular sites close to where ROS generates from the photosensitizer with light irradiation [32]. It has been previously reported that the photosensitizers localized in the cell membrane and mitochondria of cancer cells promoted the necrosis and apoptosis in PDT, respectively [33,34,35,36,37]. Due to the accumulation of AIE-M in both cell membranes and mitochondria of HeLa cells after 8 h uptake, it is possible to induce both necrosis and apoptosis after light irradiation. Annexin V-Fluorescein Isothiocyanate/Propidium Iodide (Annexin VFITC/PI) assay, western blot analysis and trypan blue staining were performed to explore the possible cell death pathway of AIE-M mediated photodynamic therapy.

**Figure 10:**
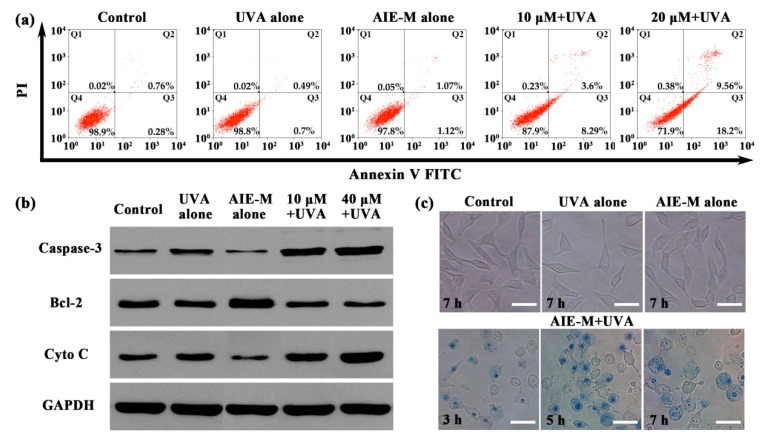
(**a**) Apoptosis of HeLa cells analyzed by flow cytometry; (**b**) Western blot analysis for the determination of the release of cytochrome c and expression of caspase-3 and Bcl-2 by the HeLa cells after PDT; (**c**) Bright-field images of HeLa cells counterstained with trypan blue. The scale bar is 40 μm.

Apoptosis is a complex, multi-step and inherent programmed cell death pathway, which is tightly regulated by various proteins. Annexin VFITC/PI staining is a classical and useful method to differentiate apoptotic and necrotic cells. HeLa cells were treated with AIE-M and 1.5 J cm^−2^ light irradiation, stained with Annexin VFITC/PI and assessed with flow cytometry analysis. The cells without any treatment severed as controls. As illustrated in Figure 10a, almost no early apoptotic and late apoptotic cells were detected in HeLa cells without any treatment, treated with AIE-M alone and light alone. When AIE-M-treated cells were irradiated with light, apoptotic cells were detected. Fixing the light dose at 1.5 J cm^−2^, the percentages of apoptotic cells were AIE-M concentration dependent. With increasing the AIE-M concentration from 10 μM to 20 μM, the percentages of early apoptotic cells increased from 8.28% to 18.19%, and those of late apoptotic cells increased from 3.6% to 9.56%. The key proteins involved in apoptosis were further detected by western blot analysis to confirm the apoptosis pathway of AIE-M-mediated PDT. Cytochrome c is one of the apoptogenic factors released from mitochondria into the cytosol, subsequently promoting apoptosis. Besides, caspase-3 is the main effector caspase that is activated in apoptosis. As shown in Figure 10b, compared with the control group without any treatment, there were no obvious differences in the expression of cytochrome c and caspase-3 in cells treated with AIE-M alone or light alone. However, the levels of cytochrome c and caspase-3 expressed in the AIE-M-treated cells together with irradiation dramatically increased with the elevation of AIE-M concentration. In contrast, the expression of the antiapoptotic Bcl-2 protein significantly decreased. The results demonstrated that apoptosis was activated in the death of HeLa cells responding to PDT with AIE-M and light irradiation.

Necrosis has been regarded as a violent and unprogrammed cell death process, which is caused by physical or chemical damage. The loss of cell membrane permeability generally occurs in necrotic cells. To confirm the involvement of necrosis in cell death induced by AIE-M-mediated PDT, cell membrane integrity was studied before and after PDT. HeLa cells were incubated with AIE-M for 8 h, and then stained with trypan blue at 3, 5, and 7 h after irradiation; meanwhile, the morphological changes of cells were also observed. As shown in Figure 10c, the cells incubated with AIE-M alone or upon irradiation alone remained in an identical morphology similar to the control cells with insignificant blue staining. However, the population of cells that stained blue increased with the extension of incubation time after PDT, which was the evidence of membrane integrity loss. Additionally, the cell membrane integrity was rapidly impaired within 3 h after irradiation, while the incidence of cell swelling, which was defined as the characteristic of necrosis, increased significantly at 5 and 7 h after PDT. Furthermore, the karyolysis, karyorrhexis, and rupture of vesicles were also observed at 5 h and enhanced at 7 h after PDT treatment, indicating the rapid necrosis of HeLa cells after PDT.

The death mechanisms induced by PDT are complex and dependent on the experimental conditions [38]. The short-term incubation of PS allows it to localize at the plasma membrane and induce necrosis-like cell death [39]. The MTT results in Figure 9c showed that the survival rate of HeLa cells incubated with AIE-M for 2 h and light irradiation decreased to 79%, indicating that the generated ROS from membrane binding AIE-M was able to induce cell death. With longer incubation times, a part of the up-taken AIE-M entered into the mitochondria of HeLa cells and destructed the organelle by ROS generated with light irradiation. Then cytochrome c released from the mitochondria into the cytoplasm, was followed by activation of the intrinsic apoptosis pathway. Meanwhile, the cell membrane was rapidly destroyed by ROS produced from cell membrane-localized AIE-M upon irradiation, resulting in disintegrated plasma membrane integrity and cell necrosis. From the trypan blue staining results (Figure 10c), it could be found that most cells were swelling at 7 h after PDT, suggesting necrosis. The possible mechanism of HeLa cell death triggered by AIE-M-mediated PDT included the combination of apoptosis from ROS generated in the mitochondria and necrosis from ROS generated in the cell membrane, and the necrosis may be a dominant cell death pathway in the early stage after PDT. 

## 4. Conclusions

In summary, we report sub-20 nm micelles (AIE-M) with AIE characteristics for image-guided PDT. AIE-M is almost nontoxic to HeLa cells and shows strong fluorescence and excellent ROS generation ability with light irradiation both in aqueous solution and in cells. When incubated with HeLa cells, AIE-M localized on the cell membrane at the early step and then gradually accumulated in the mitochondria of HeLa cells. ROS produced in the cells from AIE-M with light irradiation led to AIE-M concentration and light dose-dependent cytotoxicity. The possible mechanism of cell death triggered by AIE-M-mediated PDT included both the apoptosis and the necrosis of cancer cells from ROS generated in the mitochondria and on the cell membrane, respectively. Due to the unique advantages of AIE-M, including easy organic solvent-free preparation, high stability in aqueous solution, non-cytotoxicity, stable fluorescence emission with large Stokes shift and excellent ROS generation ability with light irradiation, AIE-M will show promising potential in cancer photodynamic therapy.

## Supplementary Materials

The following are available online at http://www.mdpi.com/2079-4991/8/11/921/s1, Scheme S1: The synthesis of AIE-1; Figure S1: ^1^H-NMR spectrum of compound 1; Figure S2: ^13^C-NMR spectrum of compound 1; Figure S3: ^1^H-NMR spectrum of compound 2; Figure S4: ^13^C-NMR spectrum of compound 2; Figure S5: ^1^H-NMR spectrum of compound 3; Figure S6: ^13^C-NMR spectrum of compound 3; Figure S7: ^1^H-NMR spectrum of AIE-1; Figure S8: ^13^C-NMR spectrum of AIE-1; Figure S9: Fluorescence lifetime decay of AIE-M in PBS; Figure S10: Change in fluorescent intensity of singlet oxygen sensor green (SOSG) (5 μM) and AIE-M (10 μM)-containing SOSG (5 μM) in PBS upon UV irradiation (365 nm) for different time.
