# Supplementary material for: Aggregation-Induced Emission (AIE) Polymeric Micelles for Imaging-Guided Photodynamic Cancer Therapy
Source: Nanomaterials (Basel). 2018 Nov 7;8(11):921. doi: 10.3390/nano8110921 (PMC6266309; doi:10.3390/nano8110921)
Supplement: Supplementary file 1 [file nanomaterials-08-00921-s001.pdf]

Supplementary materials

Aggregation-induced emission (AIE) polymeric micelles for imaging-guided photodynamic cancer therapy

Yang Zhang, Cai-Xia Wang, Shi-Wen Huang *

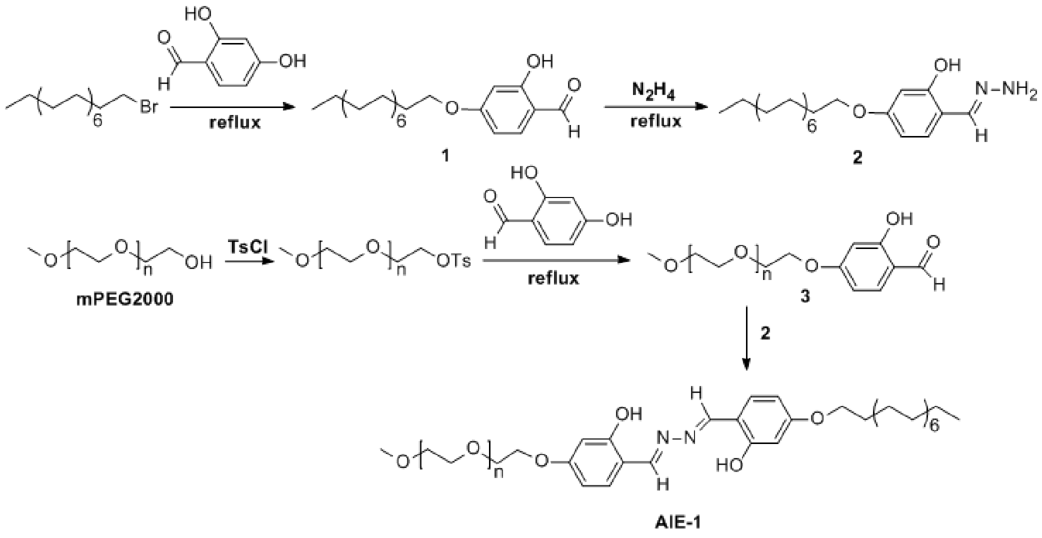

Scheme S1. Synthesis of **AIE-1**.

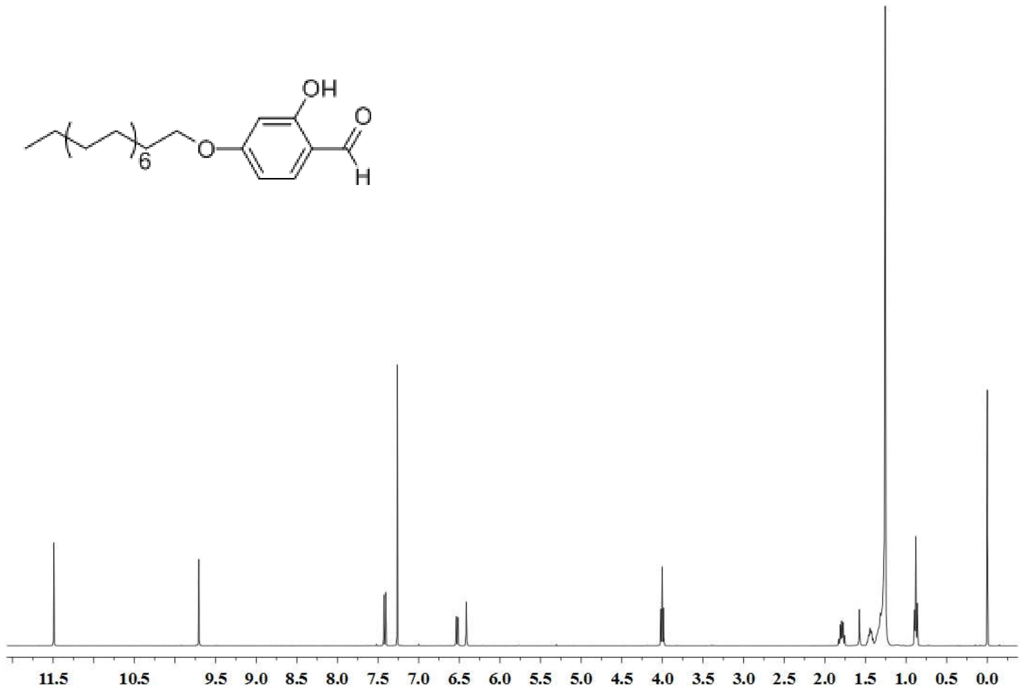

Figure S1. ^{1}H NMR spectrum of compound **1** in $CDCl_3$.

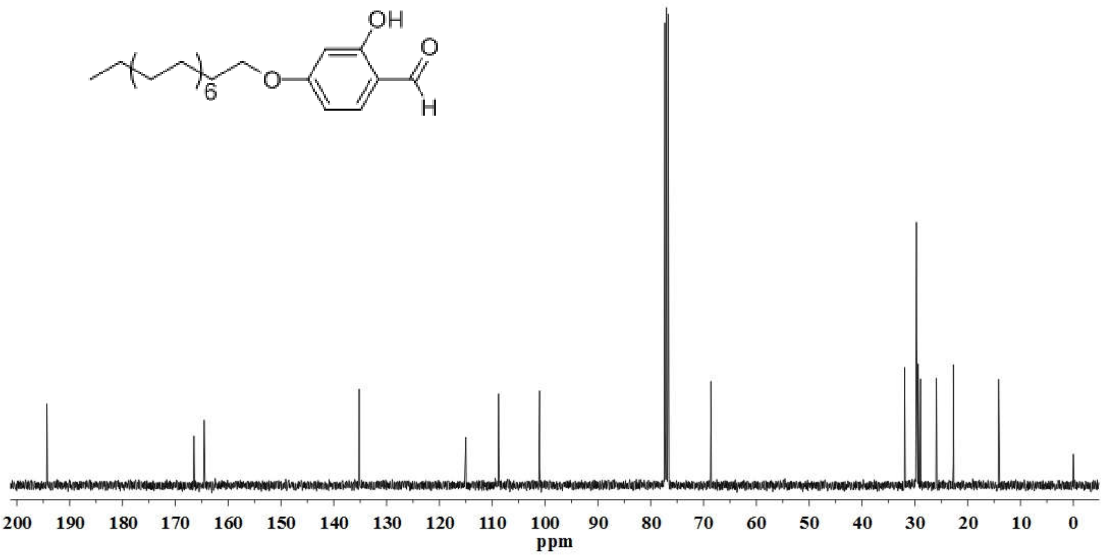

Figure S2. ^{13}C NMR spectrum of compound **1** in $CDCl_3$.

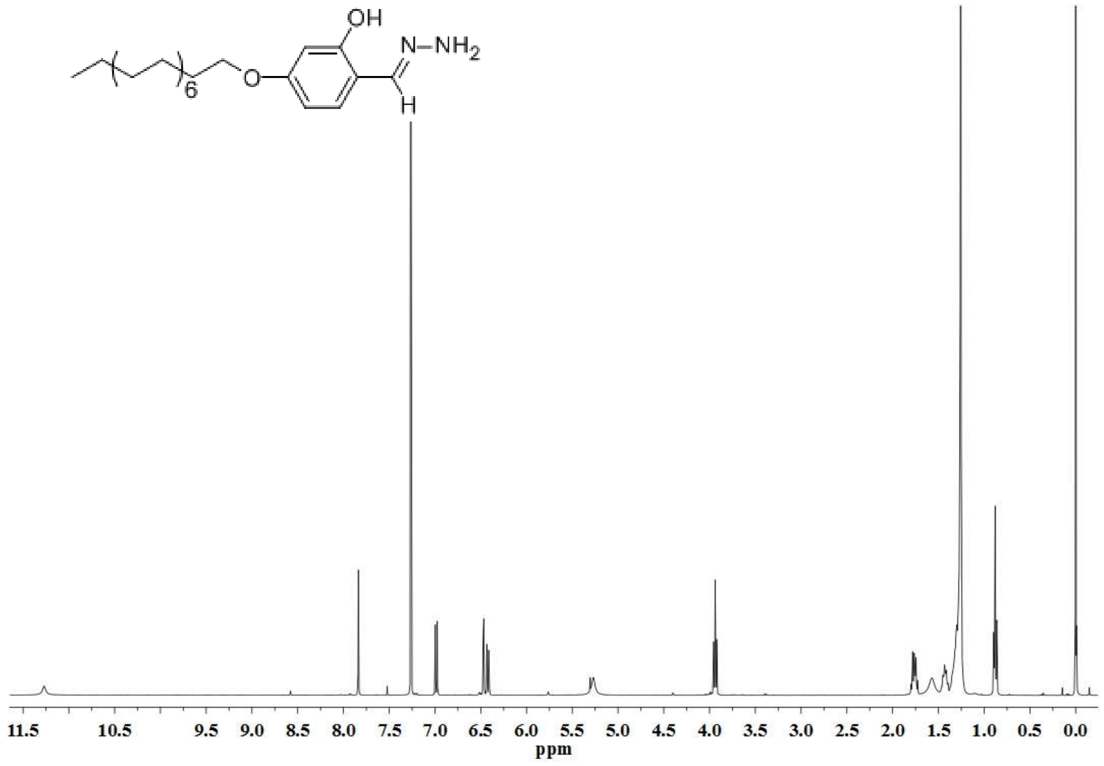

Figure S3. ^{1}H NMR spectrum of compound **2** in $CDCl_3$.

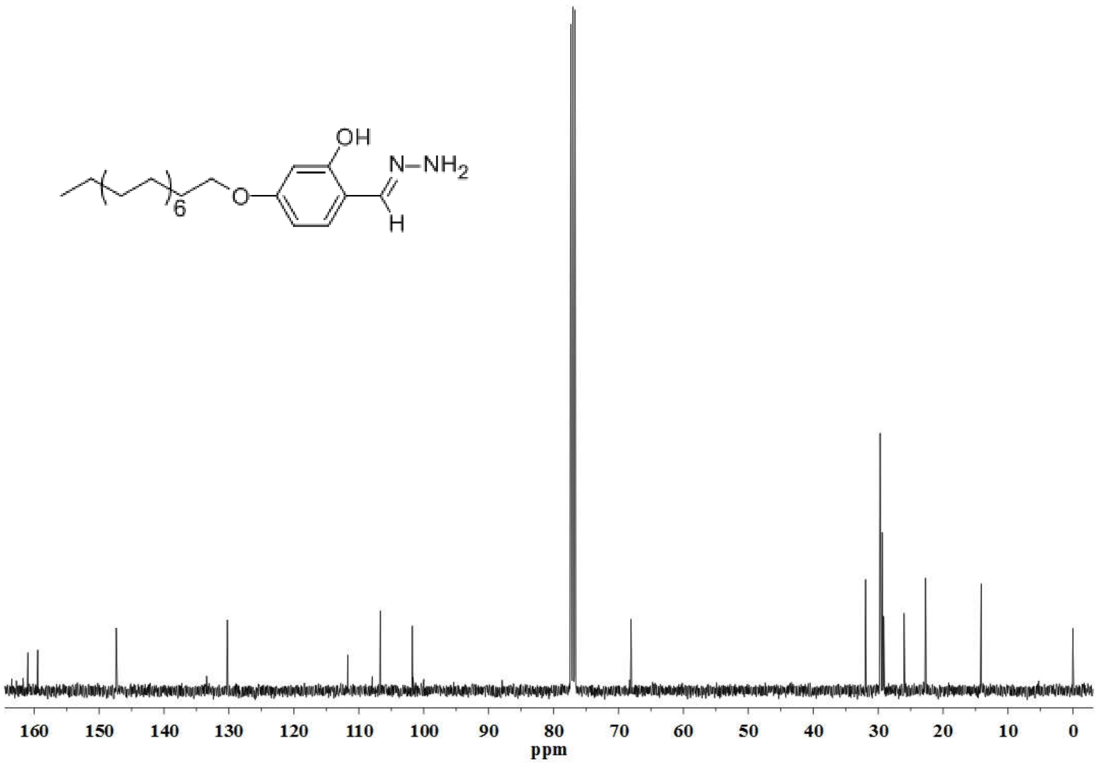

Figure S4. ^{13}C NMR spectrum of compound **2** in $CDCl_3$.

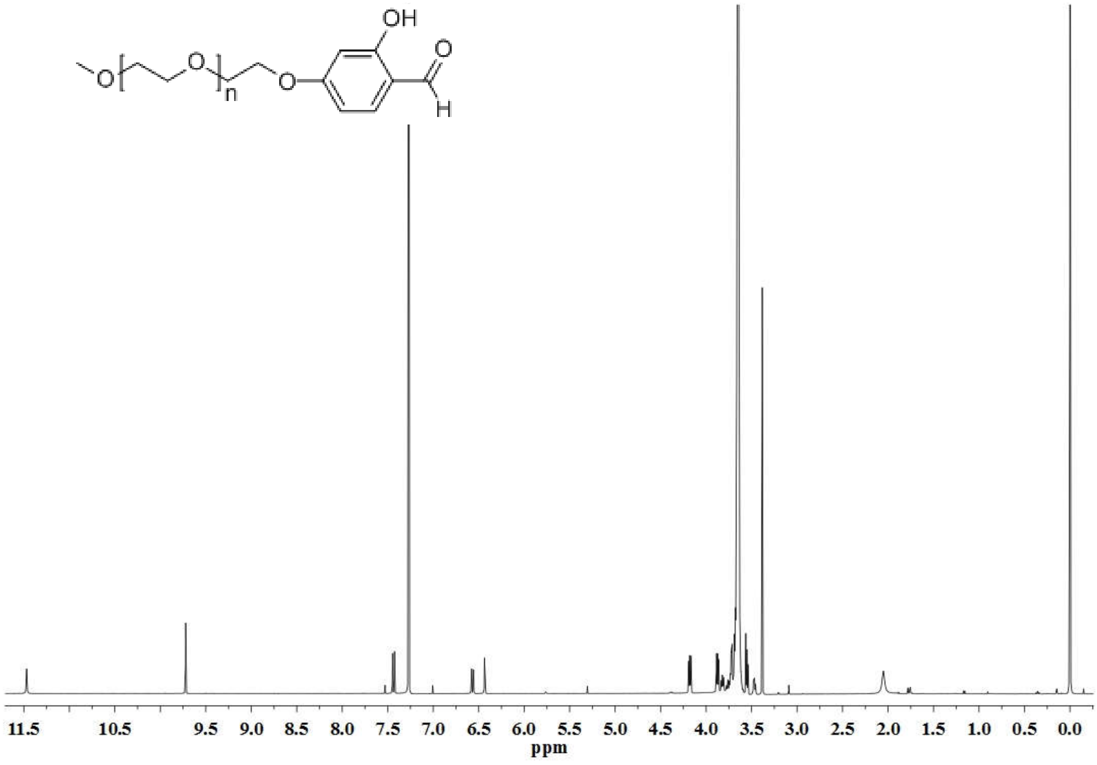

Figure S5. ^{1}H NMR spectrum of compound **3** in $CDCl_3$.

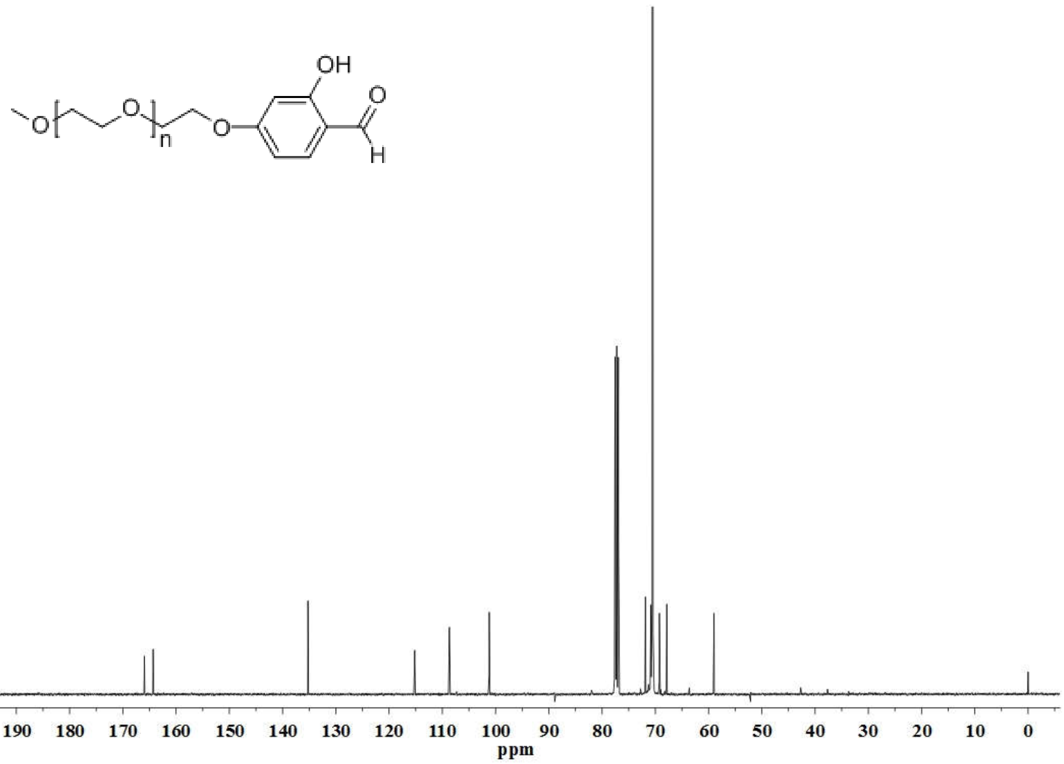

Figure S6. ^{13}C NMR spectrum of compound **3** in $CDCl_3$.

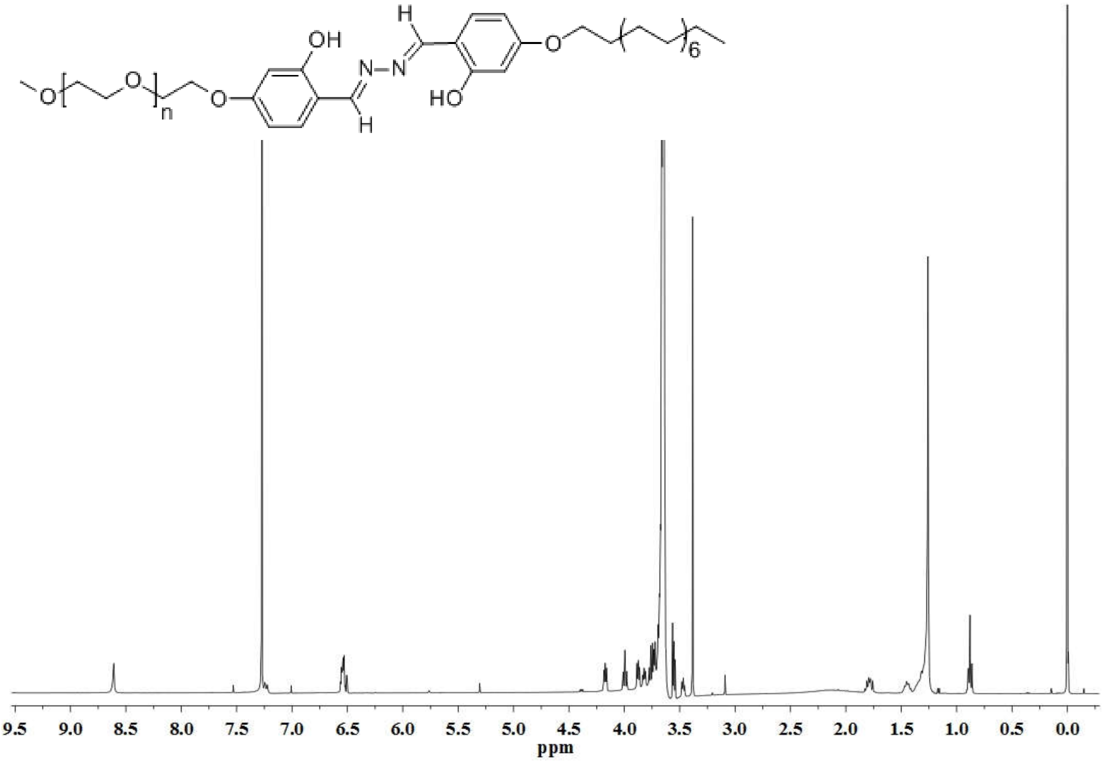

Figure S7. ^{1}H NMR spectrum of **AIE-1** in $CDCl_3$.

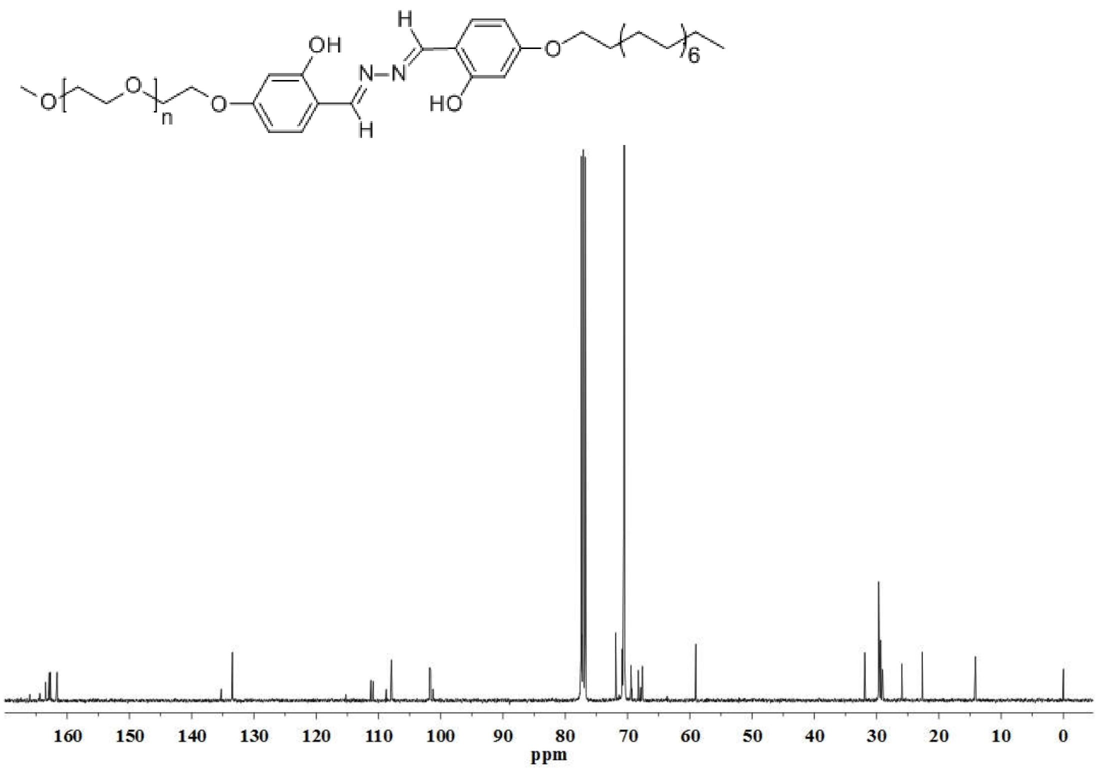

Figure S8. ^{13}C NMR spectrum of **AIE-1** in $CDCl_3$.

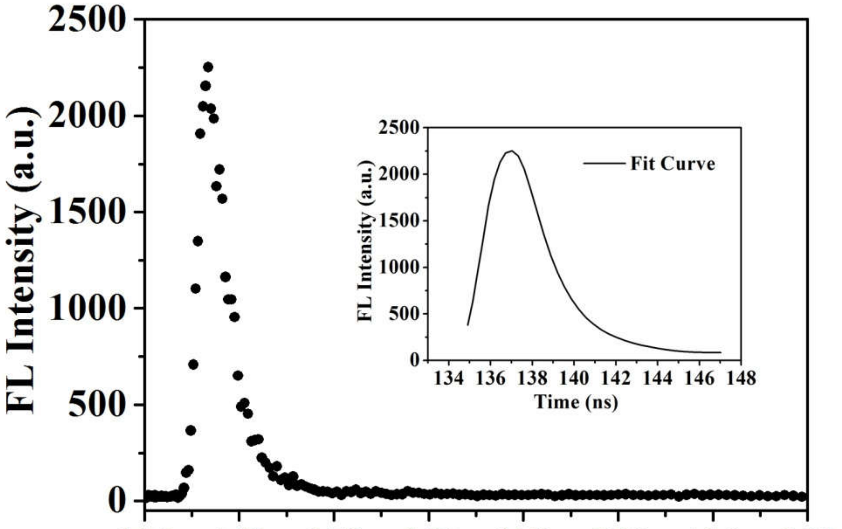

Figure S9. Fluorescence lifetime decay of **AIE-M** (20 μM) in PBS. Ex: 337 nm, Em: 525 nm.

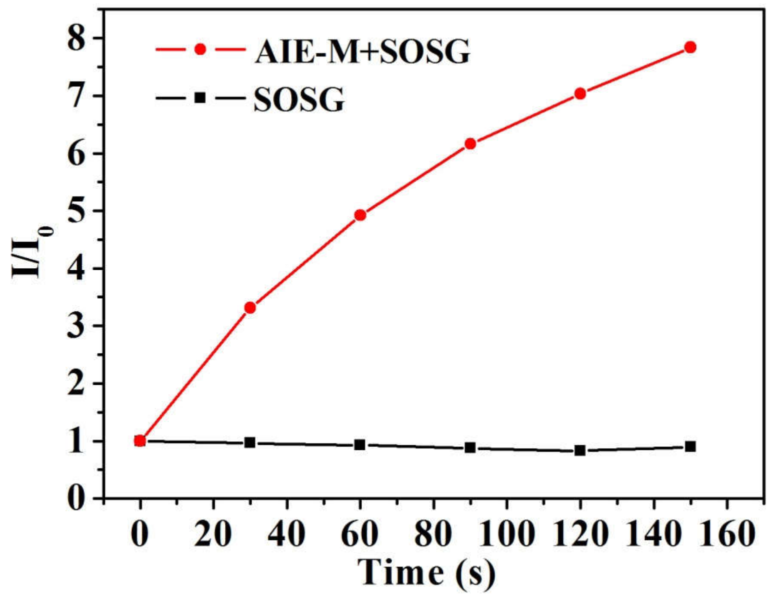

Figure S10. Change in fluorescent intensity of singlet oxygen sensor green (SOSG) (5 μM) and **AIE-M** (10 μM)-containing SOSG (5 μM) in PBS upon UV irradiation (365 nm) for different time. Ex: 488 nm, Em: 525 nm.
